# Gaussian Process Regression for Value‐Censored Functional and Longitudinal Data

**DOI:** 10.1002/sim.70277

**Published:** 2025-09-23

**Authors:** Adam Gorm Hoffmann, Claus Thorn Ekstrøm, Benjamin Zeymer Christoffersen, Andreas Kryger Jensen

**Affiliations:** ^1^ Section of Biostatistics, Department of Public Health University of Copenhagen Copenhagen Denmark; ^2^ Division of Robotics, Perception and Learning KTH Royal Institute of Technology Stockholm Sweden; ^3^ Department of Medical Epidemiology and Biostatistics Karolinska Institutet Solna Sweden

**Keywords:** Bayesian data analysis, functional data analysis, longitudinal data, outcome truncation, value‐censored data

## Abstract

Gaussian process (GP) regression is widely used for flexible and non‐parametric Bayesian modeling of data arising from underlying smooth functions. This paper introduces a solution to GP regression when the observations are subject to value‐based censoring. We derive exact and closed‐form expressions for the conditional posterior distributions of the underlying functions in both the single‐curve fitting case and in the case of a hierarchical model where multiple functions are modeled simultaneously. Our method can accommodate left, right, and interval censoring, and is directly applicable as an empirical Bayes method or integrated in a Markov–Chain Monte Carlo sampler for full posterior inference. The method is validated through extensive simulations, where it substantially outperforms naive approaches that either exclude censored observations or treat them as fully observed values. We give an application to a real‐world dataset of longitudinal HIV‐1 RNA measurements, where the observations are subject to left censoring due to a detection limit.

## 1 Introduction

Gaussian process (GP) regression is widely recognized for its flexibility and non‐parametric approach in Bayesian modeling of data derived from underlying smooth functions [1]. However, a frequent challenge encountered across various fields is the censoring of certain observations from these latent functions. This issue often arises due to detection limits, for example, in scenarios such as privacy concerns in social sciences, with light saturation in image analysis [2], or the estimation of substance concentrations from bioassays [3]. A motivating example where censored functional data is encountered in medical research is data from continuous glucose monitoring (CGM) devices that are routinely used to monitor patients with diabetes. Data from such devices are often right‐censored due to a detection limit on high glucose values. Different summary measures, such as daily average or time‐in‐range, are calculated from these CGM trajectories to characterize patients' status, and such summary measures will be biased unless the censoring is specifically modeled. Therefore, robust statistical methods to handle censoring for GP regression are essential for accurate data analysis and decision‐making.

Several authors have studied potentially left‐, right‐, or interval‐censored responses of underlying Gaussian distributions. Thiébaut and Jacqmin‐Gadda [4] reviewed and compared two likelihood‐based methods for handling left‐censored data in linear mixed models, using HIV‐1 RNA measurements as an example. Their models are fully parametric—including the random effects—and the different model specifications provide similar estimation results. However, one of their implementations allows for more flexibility of the error term, as a random process can be assumed.

The earlier paper by Hughes [5] takes an identical approach for linear mixed models but addresses the implementation problem by deriving an expectation‐maximization (EM) style solution for fast estimation in combination with a Gibbs sampler. In the paper, the author claims that a Bayesian solution might be a possible alternative, but that convergence would be prohibitively slow. The EM algorithm is also the crux of the paper by Vaida and Liu [6], where the idea of Hughes [5] is extended to non‐linear mixed effects and includes restricted maximum likelihood estimates. In addition, Vaida and Liu [6] relaxes the necessity to include Monte Carlo simulation as part of the estimation procedure, thereby obtaining a 40 times computational speedup.

Vock et al. [7] considered a mixed model framework with a smooth, semi‐nonparametric random effects density for censored longitudinal data. Their approach alleviates the problem that the maximum likelihood estimators will be inconsistent when the random‐effects density deviates substantially from normality. They find through simulations that the flexible random‐effect density reduces bias and improves efficiency compared to assuming Gaussian random effects.

Common to all of the above references is that they assume that all measurements are taken at the same (fixed) time points, and they require the random effect distribution to be specified. In addition, they are all within the frequentist framework and indirectly consider the observed and censored measurements as fixed‐time representations of an underlying process that is never modeled. We show how to remedy this and move the setting to a Bayesian framework.

Estimation of latent Gaussian processes in the presence of censoring has previously been studied by Groot and Lucas [8] and Gammelli et al. [9]. Their approaches approximate the posterior distribution of the (censored) latent process through expectation propagation since they both claim that the posterior distribution is intractable.

Mattos et al. [10] analyzes a dataset on HIV‐1 RNA levels in 46 patients where the viral load detection is censored because of the sensitivity of the lower detection limit of the laboratory assay. They propose a semiparametric mixed‐effects model with auto‐correlation and obtain estimates using the EM algorithm in a frequentist setting. Their approach is sensitive to the time registration and spacing, and their results do not provide estimates of the full latent process, let alone Bayesian interpretations. The HIV‐1 RNA data were also analyzed in a previous work by some of the same authors, where they employed a flexible longitudinal linear mixed‐effects model for censored data to compute maximum likelihood estimates [11].

In contrast, we instead give an exact representation of the conditional posterior distribution of the latent function values given the observations, the censoring limits, and the parameters governing the Gaussian process. Based on this expression, we give an exact posterior simulation procedure that, when used as part of an empirical Bayes method, gives faster computation times than full Bayesian inference via MCMC.

We provide an implementation of the method in R [12] with certain components implemented in the probabilistic programming language Stan [13] and exposed to R using the R package cmdstanr [14]. The implementation and code for applying it on the HIV‐1 RNA data set can be found on the first author's GitHub page at https://github.com/adamgorm/censored‐gp.

This manuscript is structured as follows. In Section 2, we present the hierarchical Gaussian process regression model in both the univariate case of a single latent function and in the multivariate case where a collection of latent functions is modeled together with their common mean function. We derive the likelihood functions for the observed data, the exact analytic form of the posterior distributions of the latent functions, and present a constructive way of simulating from the posterior distributions using an equivalence in distributions. In Section 3, we perform a simulation study comparing the integrated squared and absolute errors of the posteriors from our method to an oracle model and to naive ways of handling the censoring. In Section 4, we investigate frequentist coverage of the credible intervals. In Section 5, we give an application to longitudinal measurements of HIV‐1 RNA with left censoring, and we conclude with a discussion in Section 6.

*Notation*: Vectors and matrices are denoted by bold lower and upper case letters, respectively, and for vectors $x,y\in {\mathbb{R}}^{n}$ we write x>y to mean that $x_{i}>y_{i}$ for all i∈{1,…,n}. We write ϕ(·;μ,∑) and Φ(·;μ,∑) for density and cumulative density function of the multivariate normal distribution with expectation μ and covariance matrix ∑, respectively, and write P∼TN(μ,∑;l,u) when P follows a multivariate truncated normal distribution with parameters μ and ∑ truncated element‐wise from below at the vector l and above by the vector u.

## 2 Methods

### 2.1 Univariate Gaussian Process Regression

We assume a classical univariate Gaussian process regression setting with a single latent function f from which we observe noisy realizations $Y(t_{j})$. Specifically, we consider the following hierarchical Gaussian process model:

(1) $$\begin{array}{ll} \Theta & ∼G_{\Psi }\\ f|\theta & ∼\text{GP}(0,K_{\theta })\\ Y(t_{j})|f(t_{j}),\Theta \; & \overset{⫫}{∼}\;N(f(t_{j}),\sigma^{2}) \end{array}$$

with covariance function $K_{\theta }$ and some prior distribution $G_{\Psi }$ on the parameters Θ:=(θ,σ).

We will assume that at $J_{o}$ time points, $t^{o}$, we get complete observations $Y^{o}=y^{o}\in {\mathbb{R}}^{J_{o}}$, while at $J_{c}$ time points, $t^{c}$, we only observe that $Y^{c}>y^{c}\in {\mathbb{R}}^{J_{c}}$, where $y^{c}$ is a vector of censoring values. For simplicity, we only present the right‐censored case, but similar calculations would work in the left‐censored case, where we observe $Y^{c}<y^{c}$ and in the interval censored case where we observe $y^{\ell }<Y^{c}<y^{u}$. We point out that we do not put any restriction on the censoring values $y^{c}$, so in particular they are allowed to be time‐dependent. This corresponds to observing the response $\left({\tilde{Y}}_{i},\Delta_{i}\right)$ at each time $t_{i}$, where $\Delta_{i}=1$ if ${\tilde{Y}}_{i}=Y_{i}$ (indicating that at time $t_{i}$, we observed the actual response of interest $Y_{i}$) and $\Delta_{i}=0$ if ${\tilde{Y}}_{i}<Y_{i}$ (indicating that at time $t_{i}$, we only observed a censoring value ${\tilde{Y}}_{i}$ below the actual response of interest $Y_{i}$). With this notation, we define the vectors

$$\begin{array}{ll} t^{o} & =\left\{t_{i}:\Delta_{i}=1\right\},\;Y^{o}=\left\{Y_{i}:\Delta_{i}=1\right\},\;y^{o}=\{{\tilde{Y}}_{i}:\Delta_{i}=1\},\\ t^{c} & =\left\{t_{i}:\Delta_{i}=0\right\},\;Y^{c}=\left\{Y_{i}:\Delta_{i}=0\right\},\;y^{c}=\{{\tilde{Y}}_{i}:\Delta_{i}=0\} \end{array}$$

ordered identically so that $Y_{i}^{o}$ corresponds to $t_{i}^{o}$ and so on. We denote by $\tilde{t}\in {\mathbb{R}}^{J_{p}}$ the time points at which we want to evaluate the posterior of $f(\tilde{t})$ and further define the following covariance matrices according to the model specification in Equation (1) for the complete and censored data, and the latent Gaussian process as

(2) $$\begin{array}{cc} & \sum_{o}=K_{\theta }(t^{o},t^{o})+\sigma^{2}I_{J_{o}},\;\sum_{c}=K_{\theta }(t^{c},t^{c})+\sigma^{2}I_{J_{c}},\;\sum_{f}=K_{\theta }(\tilde{t},\tilde{t}),\\ & \sum_{co}=K_{\theta }(t^{c},t^{o}),\;\;\;\;\;\;\;\;\;\;\;\sum_{fc}=K_{\theta }(\tilde{t},t^{c}),\;\;\;\;\;\;\;\;\;\;\;\;\;\sum_{fo}=K_{\theta }(\tilde{t},t^{o}) \end{array}$$

where $I_{n}$ denotes the n×n identity matrix.

The goal of inference is the posterior distribution $p(f(\tilde{t})|Y^{o},Y^{c}>y^{c})$. In what follows, we will distinguish between two ways of doing posterior inference. In *full Bayesian* inference, MCMC is used to sample from this posterior, for example, using Hamiltonian Monte Carlo by implementing the posterior density $p(f(t),\Theta |Y^{o},Y^{c}>y^{c})\propto p(Y^{o},Y^{c}>y^{c}|f(t),\Theta )p(f(t)|\Theta )p(\Theta )$ (where $t=(t^{o},t^{c},\tilde{t})$) from Equation (1) in a probabilistic programming language such as Stan [13]. In *empirical Bayes* inference, instead of placing a prior $G_{\Psi }$ on Θ (see, e.g., the section *Priors for Gaussian process parameters* in [13]), a point estimate of Θ is first obtained by maximizing the log‐likelihood $\hat{\Theta }=\arg\sup_{\Theta }\log p(Y^{o}=y^{o},Y^{c}>y^{c}|\Theta )$ and then one samples from the conditional posterior $p(f(\tilde{t})|Y^{o},Y^{c}>y^{c},\hat{\Theta })$. In full Bayes one would instead have first sampled Θ from its marginal posterior $p(\Theta |Y^{o},Y^{c}>y^{c})\propto p(Y^{o},Y^{c}>y^{c}|\Theta )p(\Theta )$ and then have sampled $f(\tilde{t})$ from the conditional posterior $p(f(\tilde{t})|Y^{o},Y^{c}>y^{c},\Theta^{\left(i\right)})$ for each posterior sample $\Theta^{\left(i\right)}$ (although in practice Θ and f(t) would often be sampled jointly). This illustrates how empirical Bayes neglects uncertainty about the GP parameters Θ since they are treated as known and equal to $\hat{\Theta }$, meaning that credible intervals will tend to be narrower compared to the full Bayes posterior.

Under this model, the likelihood of the observed data is given in Proposition 1, a proof of which is given in the Supporting Information.

Proposition 1 *In the censored Gaussian process regression setting under the model in Equation* (1), *the likelihood of the observed data in terms of the parameters* Θ=(θ,σ) *is*

$$\begin{array}{ll} L(\Theta )=p(Y^{o}=y^{o}, & Y^{c}>y^{c}|\Theta )=\phi (y^{o};0_{J_{o}},\sum_{o})\Phi (-y^{c};-\xi_{c|o},\sum_{c|o}) \end{array}$$

*with* $\xi_{c|o}=\sum_{co}\sum_{o}^{-1}y_{o}$, $\sum_{c|o}=\sum_{cc}-\sum_{co}\sum_{o}^{-1}\sum_{co}^{T}$, *and the involved covariance matrices are given in Equation* (2).

The result from Proposition 1 enables us to either evaluate the likelihood in a fully Bayesian setting or to estimate the parameters Θ in an empirical Bayes setting with $\hat{\Theta }=\arg\sup_{\Theta }\log L(\Theta )$ (where one may consider reparametrizing from σ to log(σ) depending on the optimization routine used). In either case, the next step is to obtain an expression for the conditional posterior distribution $p(f(\tilde{t})|Y^{o}=y^{o},Y^{c}>y^{c},\Theta )$ such that posterior evaluations of the latent Gaussian process can be obtained at any vector of time points $\tilde{t}$. A direct calculation (given in the Supporting Information) shows that

(3) $$\begin{array}{cc} p(f(\tilde{t})|Y^{o} & =y^{o},Y^{c}>y^{c},\Theta )\\ & =\phi (f(\tilde{t})|\xi_{f|o},\sum_{f|o})\times \frac{\Phi (-y^{c};-\xi_{c|o,f},\sum_{c|o,f})}{\Phi (-y^{c};-\xi_{c|o},\sum_{c|o})} \end{array}$$

where the conditional means and covariances are given by

(4) $$\begin{array}{cc} \xi_{f|o} & =\sum_{fo}\sum_{o}^{-1}y^{o},\;\sum_{f|o}=\sum_{f}-\sum_{fo}\sum_{o}^{-1}\sum_{fo}^{T}\\ \xi_{c|o} & =\sum_{co}\sum_{o}^{-1}y_{o},\;\sum_{c|o}=\sum_{cc}-\sum_{co}\sum_{o}^{-1}\sum_{co}^{T}\\ \xi_{c|o,f} & =\left(\begin{array}{cc} \sum_{co} & \sum_{cf} \end{array}\right){\left(\begin{array}{cc} \sum_{o} & \sum_{of}\\ \sum_{fo} & \sum_{f} \end{array}\right)}^{-1}\left(\begin{array}{c} y^{o}\\ f(\tilde{t}) \end{array}\right)\\ \sum_{c|o,f} & =\sum_{c}-\left(\begin{array}{cc} \sum_{co} & \sum_{cf} \end{array}\right){\left(\begin{array}{cc} \sum_{o} & \sum_{of}\\ \sum_{fo} & \sum_{f} \end{array}\right)}^{-1}\left(\begin{array}{c} \sum_{co}^{T}\\ \sum_{cf}^{T} \end{array}\right) \end{array}$$

based on the different evaluations of the covariance kernel defined in Equation (2).

It is of interest to note that the conditional posterior density in Equation (3) consists of the term $\phi (f(\tilde{t})|\xi_{f|o},\sum_{f|o})$, which is the posterior distribution for only the non‐censored observations, scaled by the term $\Phi (-y^{c};-\xi_{c|o,f},\sum_{c|o,f})\Phi {\left(-y^{c};-\xi_{c|o},\sum_{c|o}\right)}^{-1}$, which takes the censored observations into account. This scaling term gives higher weight to functions f that take on large values at the censoring times.

As the form of the conditional posterior density is non‐standard, it is not immediately clear how to draw samples from it. Using a result from Arellano‐Valle et al. [15, eqn. (15)], Proposition 2 states a constructive way for simulating random variables from a distribution having a density function of the form given in Equation (3). A proof is given in the Supporting Information.

Proposition 2 *The distribution of* $f(\tilde{t})|Y^{o}=y^{o},Y^{c}>y^{c},\Theta$ *is the same as the distribution of* $\xi_{f|o}+\sum_{fc|o}\sum_{c|o}^{-1}P+Q$, *where*

$$\begin{array}{ll} P & ∼TN(0_{J_{c}},\sum_{c|o},y^{c}-\xi_{c|o},\infty ),\\ Q & ∼N\left(0_{J_{p}},\sum_{f|o}-\sum_{fc|o}\sum_{c|o}^{-1}\sum_{fc|o}^{T}\right) \end{array}$$

*with* $\sum_{fc|o}=\sum_{fc}-\sum_{fo}\sum_{o}^{-1}\sum_{oc}$.

From the result in Proposition 2, the conditional posterior distribution of the latent Gaussian process, f, at any vector of evaluation points $\tilde{t}$ can be simulated by drawing from the corresponding multivariate normal and multivariate truncated normal distributions and combining them according to the expression. It thus eliminates the need for potentially inefficient custom MCMC procedures to sample from the density in Proposition 1, instead requiring just an efficient way to simulate from the multivariate truncated normal distribution, which is already implemented in, for example, the TruncatedNormal package by Botev and Belzile [16]. This can either be implemented in the empirical Bayes setting where Θ is substituted by $\hat{\Theta }$ that maximizes the likelihood in Proposition 1 or in the fully Bayesian setting with posterior draws of Θ using a Markov chain Monte Carlo algorithm. A simulation study included in the Supporting Information shows that an empirical Bayes implementation using Proposition 2 is faster than a naive full Bayes MCMC implementation and that the relative speed difference increases with the number of observations and number of points to predict at. It is, for example, about 19 times faster when fitting to 500 time points and predicting at 500 time points.

In the case of left censoring or interval censoring, similar applications of Arellano‐Valle et al. [15, eqn. (15)] give ways to simulate from the conditional posterior distribution as in Proposition 2. For left‐censored data (i.e., $Y^{c}<y^{c}$) one must draw $P∼TN(0_{J_{c}},\sum_{c|o},-\infty ,y^{c}-\xi_{c|o})$ and for interval‐censored data (i.e., $y^{\ell }<Y^{c}<y^{u}$) one must draw $P∼TN(0_{J_{c}},\sum_{c|o},y^{\ell }-\xi_{c|o},y^{u}-\xi_{c|o})$.

### 2.2 Multi‐Level Gaussian Process Regression

We now turn to the multi‐level Gaussian process regression setting studied in Kaufman and Sain [17] and Hoffmann et al. [18] and show how to include censoring with calculations very similar to those for a single observed function given above. This model extends Gaussian process regression to a setting with multiple observed functions $f_{i}=\mu +\eta_{i}$ which are given by a common mean function μ and subject‐specific deviations $\eta_{i}$, both of which are modeled with Gaussian process priors. To avoid the identifiability issue that arises due to modeling the n functions $f_{1},\ldots ,f_{n}$ with the n+1 functions $\mu ,\eta_{1},\ldots ,\eta_{n}$, the $\eta_{i}$ are required to sum to zero. This is achieved by specifying dependent Gaussian proces priors with covariance kernel $K_{\theta_{\eta }}^{\eta }$ and cross‐covariances given by $\text{Cov}(\eta_{i}(t_{i}),\eta_{j}(t_{j})|\theta_{\eta })=-\frac{1}{n-1}K_{\theta_{\eta }}^{\eta }(t_{i},t_{j})$ for i≠j. Writing ${\text{GP}}_{n}^{\text{sum‐to‐zero}}$ for the joint dependent Gaussian process prior just described and $\Theta :=(\theta_{\mu },\theta_{\eta },\sigma )$, the model can be written

(5) $$\begin{array}{ll} \Theta & ∼G_{\Psi }\\ \mu |\theta_{\mu } & ∼\text{GP}\left(0,K_{\theta_{\mu }}^{\mu }\right)\\ (\eta_{1},\ldots ,\eta_{n})|\theta_{\eta } & ∼{\text{GP}}_{n}^{\text{sum}‐\text{to}‐\text{zero}}\left(0,K_{\theta_{\eta }}^{\eta }\right)\\ f_{i} & =\mu +\eta_{i}\\ y_{i}(t_{ij})|f_{i},\Theta & \overset{⫫}{∼}N\left(f_{i}(t_{ij}),\sigma^{2}\right) \end{array}$$

where $G_{\Psi }$ is some prior distribution.

We now have n latent functions $f_{i}=\mu +\eta_{i}$ from which we get noisy observations. We write $Y^{o}={\left({\left(Y_{1}^{o}\right)}^{T},\ldots ,{\left(Y_{n}^{o}\right)}^{T}\right)}^{T}$ for the noisy non‐censored realizations at the $J_{o}=\sum_{i=1}^{n}J_{i}^{o}$ times $t^{o}={\left({\left(t_{1}^{o}\right)}^{T},\ldots ,{\left(t_{n}^{o}\right)}^{T}\right)}^{T}$ and $Y^{c}={\left({\left(Y_{1}^{c}\right)}^{T},\ldots ,{\left(Y_{n}^{c}\right)}^{T}\right)}^{T}$ for the noisy censored observations at the $J_{c}=\sum_{i=1}^{n}J_{i}^{c}$ times $t^{c}={\left({\left(t_{1}^{c}\right)}^{T},\ldots ,{\left(t_{n}^{c}\right)}^{T}\right)}^{T}$. We fully observe $Y^{o}=y^{o}$, while we only see that $Y^{c}>y^{c}$. No censoring mechanism is imposed, so in particular, the censoring values can differ among the latent functions or be time‐dependent. The model definition in Equation (5) gives the relevant covariance matrices

(6) $$\begin{array}{cc} \Omega_{o_{i,j}} & :=\text{Cov}(Y_{i}^{o},Y_{j}^{o}|\Theta )\\ & =\left\{\begin{array}{ll} K_{\theta_{\mu }}^{\mu }(t_{i}^{o},t_{i}^{o})+K_{\theta_{\eta }}^{\eta }(t_{i}^{o},t_{i}^{o})+\sigma^{2}I_{J_{i}^{o}},\; & i=j\\ K_{\theta_{\mu }}^{\mu }(t_{i}^{o},t_{j}^{o})-\frac{1}{n-1}K_{\theta_{\eta }}^{\eta }(t_{i}^{o},t_{j}^{o}),\; & i\neq j \end{array}\right.\\ \Omega_{c_{i,j}} & :=\text{Cov}(Y_{i}^{c},Y_{j}^{c}|\Theta )\\ & =\left\{\begin{array}{ll} K_{\theta_{\mu }}^{\mu }(t_{i}^{c},t_{i}^{c})+K_{\theta_{\eta }}^{\eta }(t_{i}^{c},t_{i}^{c})+\sigma^{2}I_{J_{i}^{c}},\; & i=j\\ K_{\theta_{\mu }}^{\mu }(t_{i}^{c},t_{j}^{c})-\frac{1}{n-1}K_{\theta_{\eta }}^{\eta }(t_{i}^{c},t_{j}^{c}),\; & i\neq j \end{array}\right.\\ \Omega_{co_{i,j}} & :=\text{Cov}(Y_{i}^{c},Y_{j}^{o}|\Theta )\\ & =\left\{\begin{array}{ll} K_{\theta_{\mu }}^{\mu }(t_{i}^{c},t_{i}^{o})+K_{\theta_{\eta }}^{\eta }(t_{i}^{c},t_{i}^{o}),\; & i=j\\ K_{\theta_{\mu }}^{\mu }(t_{i}^{c},t_{j}^{o})-\frac{1}{n-1}K_{\theta_{\eta }}^{\eta }(t_{i}^{c},t_{j}^{o}),\; & i\neq j \end{array}\right.\\ \Omega_{\mu } & :=\text{Cov}(\mu (\tilde{t})|\Theta )\\ & =K_{\theta_{\mu }}^{\mu }(\tilde{t},\tilde{t}),\;\Omega_{\mu c}:=\text{Cov}(\mu (\tilde{t}),Y^{c}|\Theta )=K_{\theta_{\mu }}^{\mu }(\tilde{t},t^{c}),\\ \Omega_{\mu o} & :=\text{Cov}(\mu (\tilde{t}),Y^{o}|\Theta )=K_{\theta_{\mu }}^{\mu }(\tilde{t},t^{o}). \end{array}$$

Using these covariance matrices, Proposition 3 gives the likelihood of the observed data. A proof is given in the Supporting Information.

Proposition 3 *In the censored Gaussian process regression setting under the model in Equation *(5), *the likelihood of the observed data in terms of the parameters* $\Theta =(\theta_{\mu },\theta_{\eta },\sigma )$ *is*

$$\begin{array}{cc} L(\Theta ) & =p(Y^{o}=y^{o},Y^{c}>y^{c}|\Theta )\\ & =\phi (y^{o};0_{J_{o}},\Omega_{o})\Phi (-y^{c};-\nu_{c|o},\Omega_{c|o}) \end{array}$$

*with* $\nu_{c|o}=\Omega_{co}\Omega_{o}^{-1}y^{o}$ *and* $\Omega_{c|o}=\Omega_{c}-\Omega_{co}\Omega_{o}^{-1}\Omega_{co}^{T}$.

Similar to the univariate Gaussian process regression setting above, the next step is to find an expression for the conditional posterior distribution $\mu (\tilde{t}),\eta (\tilde{t})|Y^{o}=y^{o},Y^{c}>y^{c},\Theta$, which will also give us the conditional posterior distribution of each $f_{i}(\tilde{t})=\mu (\tilde{t})+\eta_{i}(\tilde{t})$. To derive the density, we have to consider $\eta^{\prime }(\tilde{t}):={\left(\eta_{1}{\left(\tilde{t}\right)}^{T},\ldots ,\eta_{n-1}{\left(\tilde{t}\right)}^{T}\right)}^{T}$ where we exclude $\eta_{n}$ because it is completely determined from $\eta_{1},\ldots ,\eta_{n-1}$ since they must sum to zero and would thus lead to a degenerate normal distribution without density. A calculation similar to the univariate setting and given in the Supporting Information, gives the conditional joint posterior density

(7) $$\begin{array}{cc} p(\mu (\tilde{t}),\eta^{\prime }(\tilde{t})|Y^{o} & =y^{o},Y^{c}>y^{c},\Theta )\\ & =\phi (\mu (\tilde{t}),\eta^{\prime }(\tilde{t})|\nu_{\mu ,\eta^{\prime }|o},\Omega_{\mu ,\eta^{\prime }|o})\times \frac{\Phi (-y^{c};-\nu_{c|\mu ,\eta^{\prime },o},\Omega_{c|\mu ,\eta^{\prime },o})}{\Phi (-y^{c};-\nu_{c|o},\Omega_{c|o})} \end{array}$$

where

(8) $$\begin{array}{ccc} \nu_{\mu ,\eta^{\prime }|o} & =\left(\begin{array}{c} \Omega_{\mu o}\\ \Omega_{\eta^{\prime }o} \end{array}\right)\Omega_{o}^{-1}y^{o}, & \\ \Omega_{\mu ,\eta^{\prime }|o} & =\left(\begin{array}{cc} \Omega_{\mu } & 0_{J_{p},(n-1)J_{p}}\\ 0_{(n-1)J_{p},J_{p}} & \Omega_{\eta^{\prime }} \end{array}\right)-\left(\begin{array}{c} \Omega_{\mu o}\\ \Omega_{\eta^{\prime }o} \end{array}\right)\Omega_{o}^{-1}\left(\begin{array}{cc} \Omega_{o\mu } & \Omega_{o\eta^{\prime }} \end{array}\right) & \\ \nu_{c|\mu ,\eta^{\prime },o} & =\left(\begin{array}{ccc} \Omega_{c\mu } & \Omega_{c\eta^{\prime }} & \Omega_{co} \end{array}\right){\left(\begin{array}{ccc} \Omega_{\mu } & 0_{J_{p},(n-1)J_{p}} & \Omega_{\mu o}\\ 0_{(n-1)J_{p},J_{p}} & \Omega_{\eta^{\prime }} & \Omega_{\eta^{\prime }o}\\ \Omega_{o\mu } & \Omega_{o\eta^{\prime }} & \Omega_{o} \end{array}\right)}^{-1}\left(\begin{array}{c} \mu (\tilde{t})\\ \eta^{\prime }(\tilde{t})\\ y^{o} \end{array}\right) & \\ \Omega_{c|\mu ,\eta^{\prime },o} & =\Omega_{c}-\left(\begin{array}{ccc} \Omega_{c\mu } & \Omega_{c\eta^{\prime }} & \Omega_{co} \end{array}\right){\left(\begin{array}{ccc} \Omega_{\mu } & 0_{J_{p},(n-1)J_{p}} & \Omega_{\mu o}\\ 0_{(n-1)J_{p},J_{p}} & \Omega_{\eta^{\prime }} & \Omega_{\eta^{\prime }o}\\ \Omega_{o\mu } & \Omega_{o\eta^{\prime }} & \Omega_{o} \end{array}\right)}^{-1} & \\ & \;\times \left(\begin{array}{c} \Omega_{\mu c}\\ \Omega_{\eta^{\prime }c}\\ \Omega_{oc} \end{array}\right) & \end{array}$$

Analogous to the univariate setting, the density of the conditional joint posterior distribution consists of a term that only includes the observed values scaled by a term involving the censored values. Again using the result from Arellano‐Valle et al. [15, eqn. (15)], Proposition 4 gives a way of sampling from this non‐standard density relying on the covariance matrices

(9) $$\begin{array}{ccc} \Omega_{(\mu ,\eta^{\prime })c|o} & =\left(\begin{array}{c} \Omega_{\mu c}\\ \Omega_{\eta^{\prime }c} \end{array}\right)-\left(\begin{array}{c} \Omega_{\mu o}\Omega_{o}^{-1}\Omega_{oc}\\ \Omega_{\eta^{\prime }o}\Omega_{o}^{-1}\Omega_{oc} \end{array}\right) & \\ \Omega_{\mu c} & =K_{\theta_{\mu }}^{\mu }(\tilde{t},t^{c}) & \\ \Omega_{\mu o} & =K_{\theta_{\mu }}^{\mu }(\tilde{t},t^{o}) & \\ \Omega_{{\eta^{\prime }c}_{i,j}} & =\left\{\begin{array}{ll} K_{\theta_{\eta }}^{\eta }(\tilde{t},t_{i}^{c}),\; & i=j\\ -\;\frac{1}{n-1}K_{\theta_{\eta }}^{\eta }(\tilde{t},t_{j}^{c}),\; & i\neq j \end{array}\right. & \\ \Omega_{{\eta^{\prime }o}_{i,j}} & =\left\{\begin{array}{ll} K_{\theta_{\eta }}^{\eta }(\tilde{t},t_{i}^{o}),\; & i=j\\ -\;\frac{1}{n-1}K_{\theta_{\eta }}^{\eta }(\tilde{t},t_{j}^{o}),\; & i\neq j \end{array}\right. & \end{array}$$

that follow from the model specification in Equation (5). The proof can be found in the Supporting Information.

Proposition 4 *The conditional posterior* ${\left(\mu {\left(\tilde{t}\right)}^{T},\eta^{\prime }{\left(\tilde{t}\right)}^{T}\right)}^{T}|Y^{o}=y^{o},Y^{c}>y^{c},\Theta$ *is the same as the distribution of*

$$\nu_{\mu ,\eta^{\prime }|o}+\Omega_{(\mu ,\eta^{\prime })c|o}\Omega_{c|o}^{-1}P_{\mu ,\eta^{\prime }}+Q_{\mu ,\eta^{\prime }}$$

*where* $P_{\mu ,\eta^{\prime }}$ *is truncated normal* $TN(0_{J_{c}},\Omega_{c|o},y^{c}-\nu_{c|o},\infty )$ *truncated at the lower bound* $y^{c}-\nu_{c|o}$, *and* $Q_{\mu ,\eta^{\prime }}∼N(0_{nJ_{p}},\Omega_{\mu ,\eta^{\prime }|o}-\Omega_{(\mu ,\eta^{\prime })c|o}\Omega_{c|o}^{-1}{\left(\Omega_{(\mu ,\eta^{\prime })c|o}\right)}^{T})$.

Proposition 4 thus shows how to transform draws of multivariate normal and multivariate truncated normal variables to draws from the conditional posterior distribution in Equation (7).

## 3 Simulation Study

**FIGURE 1:**
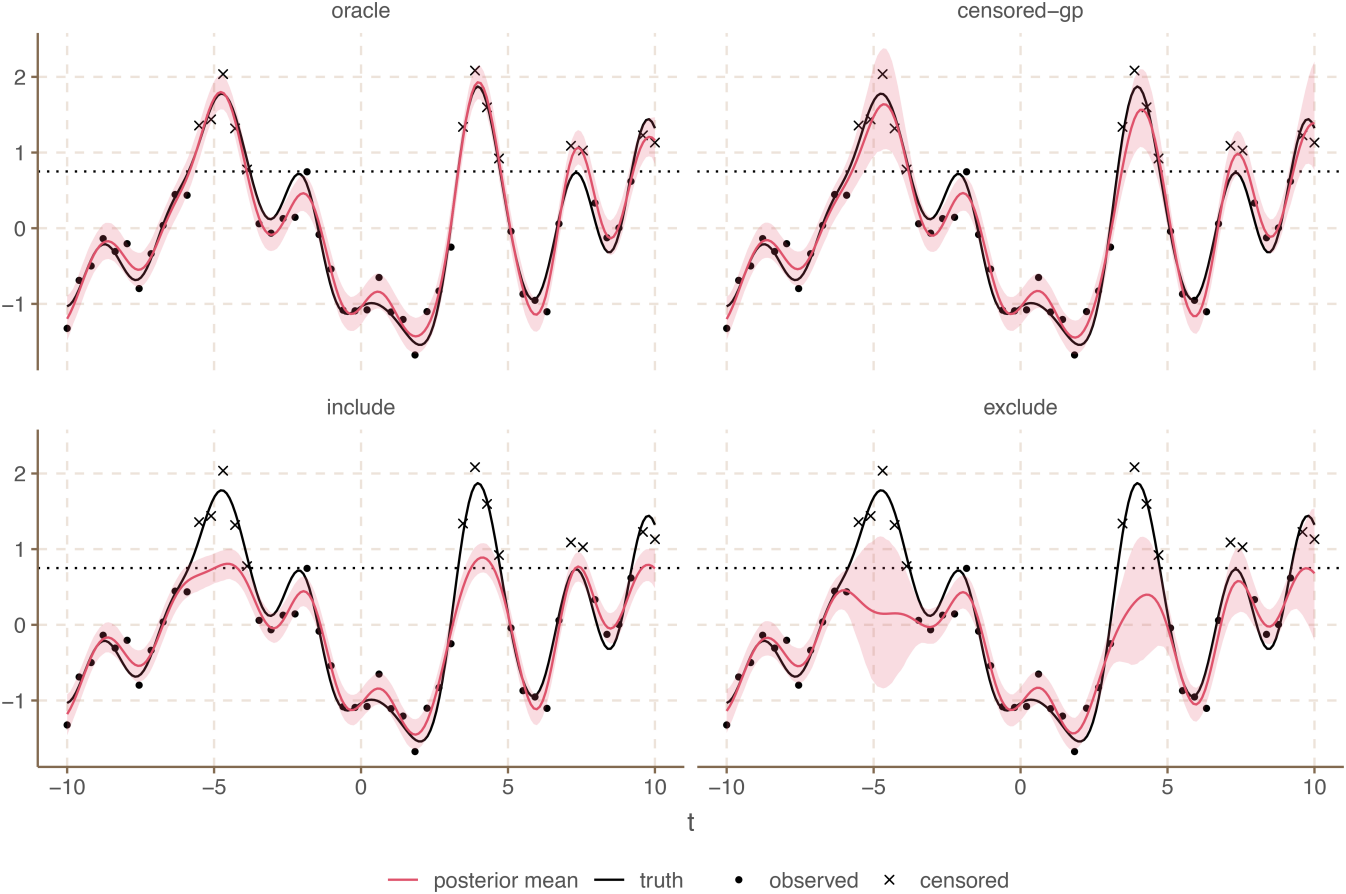
Comparison of the conditional posteriors for different Gaussian process regression methods fitted on simulated censored data. We have drawn a latent function f (black line) from a GP and take 50 equidistant observations between −10 and 10 with iid. Gaussian noise. All observations above 0.75 (dotted line) are censored. Observations are shown as points and censored observations as crosses. We show conditional posterior means (red line) and pointwise 90% credible intervals (red ribbon) for the latent function f using four different methods: The oracle method that gets to see the censored observations, our proposed censored‐GP model that correctly accounts for censoring, the include method that includes the censoring values as observations, and the exclude method that excludes all censored observations. Both include and exclude lead to bias.

In Figure 1, we show the conditional posterior of f obtained by using the model above on simulated data. We compare it to the oracle method, where we have access to all censored observations and to two naive approaches: The include method, where we include the censoring values as observations, and the exclude method, where we completely exclude all censored observations. We have drawn f from a Gaussian process with the exponentiated quadratic covariance kernel with magnitude 1 and length scale 1. At 50 equidistant points between −10 and 10, we take an observation of f and add iid. $N(0,0.2^{2})$ noise. We censor all observations above 0.75. To fit the posterior, we use empirical Bayes, where we first maximize the log‐likelihood to get a point estimate of the kernel parameters and then use Proposition 2 to draw 1000 samples of $f(\tilde{t})$ from the conditional posterior given the point estimate of the kernel parameters, where $\tilde{t}$ is a fine equidistant grid of 200 time points. In this example, including or excluding the censoring values as observations leads to bias, since it doesn't correctly describe the censored parts of the latent function. Our censored‐GP model, however, gives a conditional posterior distribution that also follows the censored parts of the latent function, albeit with wider credible bands than if no observations had been censored.

**FIGURE 2:**
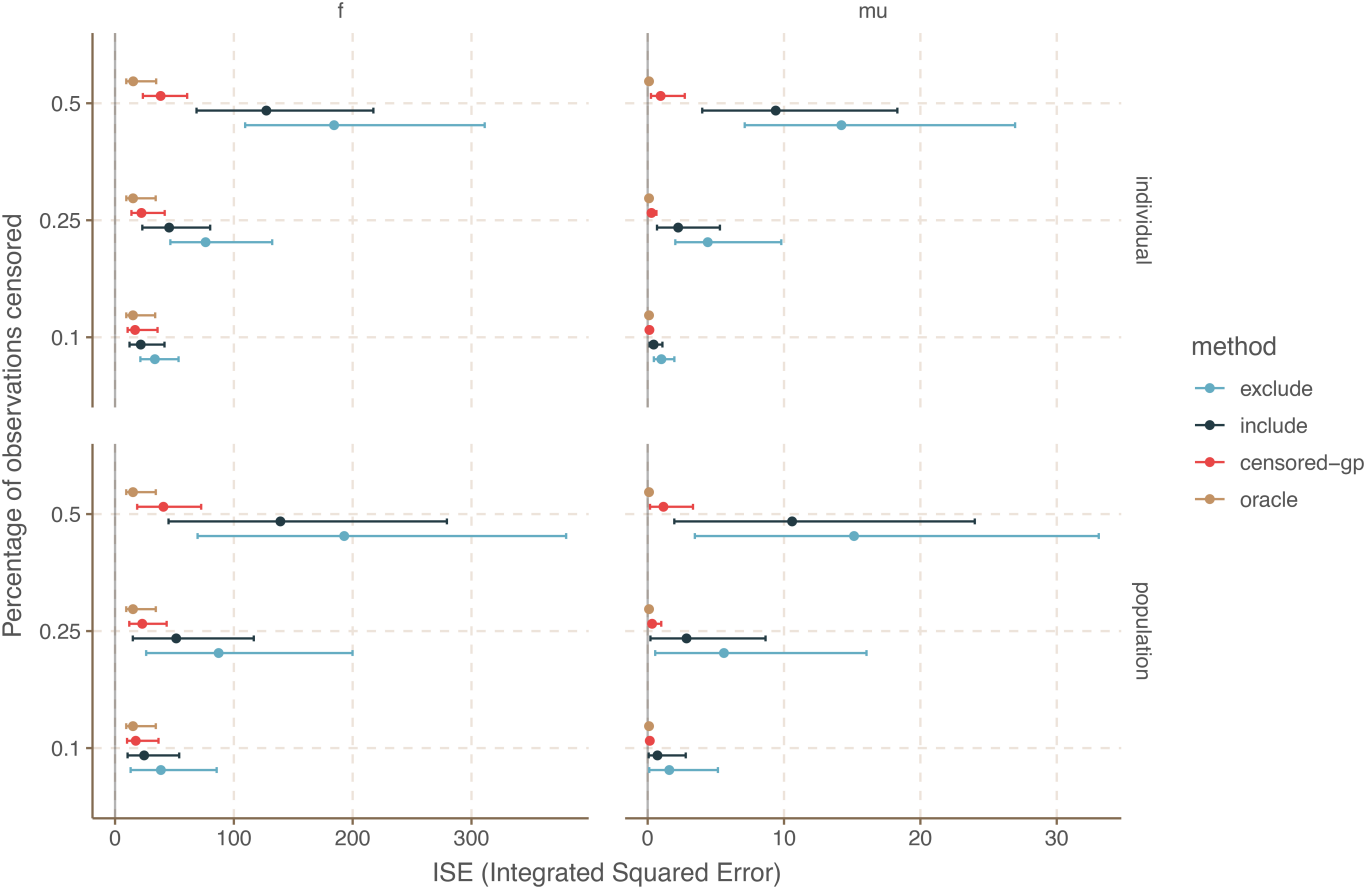
The performance of our proposed method for value‐censored Gaussian process regression compared against that of an oracle method and two baseline methods on 1000 simulated data sets observed at $n_{\text{obs}}=26$ equidistant time points. We compare performance when fitting both the latent curves $f_{1},\ldots ,f_{10}$ and their common mean‐process μ and quantify performance by computing the integrated squared error (ISE) of the conditional posterior mean compared to the truth: Smaller is better. This is done for two censoring schemes: The individual censoring scheme, where a different censoring cut‐off value is chosen for each data set so that each obtains the desired censoring percentage, and the population censoring scheme, where a single censoring cut‐off value is chosen to obtain the desired total censoring percentage across all 1000 data sets. The plot shows the mean (point) and 90% intervals (bar) across the 1000 simulated data sets.

Having illustrated how our method performs compared to naive methods and the oracle in a single example, we now do a larger simulation study to compare their performance in the multi‐level setting with 10 latent functions. We do this by comparing the integrated squared error (ISE) of the conditional posterior means of μ and $f_{1},\ldots ,f_{10}$ from the different methods on 1000 simulated data sets. The Supporting Information also provides results for the integrated absolute error (IAE) and the sup‐norm difference. Means and 90% intervals for the ISE of the four methods across the 1000 data sets are shown in Figure 2. The smaller these are, the better the conditional posterior means match the true underlying μ and $f_{1},\ldots ,f_{10}$ on the simulated data sets. Our method, which correctly accounts for censoring, is seen to outperform both the exclude and include methods and gets much closer to the performance of the oracle method. We refer to the Supporting Information for other simulation settings and error measures.

The details of the simulation setup are as follows. We simulate 1000 data sets from the setting with n=10 curves, where μ comes from a GP with exponentiated quadratic kernel with magnitude 1 and length scale 1, and η comes from a sum‐to‐zero GP with exponentiated quadratic kernel with magnitude 0.5 and length scale 0.5. This gives us the 10 latent curves $f_{i}=\mu +\eta_{i}$, i∈{1,…,10}. We observe each curve at $n_{\text{obs}}=26$ equidistant time points between −10 and 10 and add iid. $N(0,0.2^{2})$ noise; see the Supporting Information for simulations with $n_{\text{obs}}=51$. A censoring cut‐off above which all observations are censored is chosen to achieve a desired censoring percentage $p_{\text{cens}}$ in two ways: A different censoring cut‐off for each of the 1000 data sets so that each has $p_{\text{cens}}$ of its observations censored and the same cut‐off for all 1000 data sets so that all together $p_{\text{cens}}$ of the observations are censored across all 1000 data sets. To fit the posterior for each data set, we use empirical Bayes, where we first maximize the log‐likelihood to get a point estimate of the kernel parameters. We then use Proposition 4 to draw 100 samples of $f(\tilde{t})$ from the conditional posterior given the point estimate of the kernel parameters, where $\tilde{t}$ is a fine equidistant grid of 201 time points. We do this for each of the 1000 data sets. For each data set number i∈{1,…,1000} we calculate posterior means ${\bar{\mu }}^{i}$ and ${\bar{f}}_{j}^{i}$ and compute ISE, IAE and the sup‐norm distance (SUP) for both μ and f as follows. For each posterior mean ${\bar{\mu }}^{i}(\tilde{t})$ we use trapezoidal integration to approximate ${\text{ISE}}_{\mu }^{i}=\int_{-10}^{10}{\left(\mu (t)-{\bar{\mu }}^{i}(t)\right)}^{2}dt$ and ${\text{IAE}}_{\mu }^{i}=\int_{-10}^{10}|\mu (t)-{\bar{\mu }}^{i}(t)|dt$ and calculate ${\mathrm{SUP}}_{\mu }^{i}=\max_{t\in \tilde{t}}|\mu (t)-{\bar{\mu }}^{i}(t)|$. For each posterior mean ${\bar{f}}^{i}(\tilde{t})$ we use trapezoid integration to approximate ${\text{ISE}}_{f}^{i}=\sum_{j=1}^{10}\int_{-10}^{10}{\left(f_{j}(t)-{\bar{f}}_{j}^{i}(t)\right)}^{2}dt$ and ${\text{IAE}}_{f}^{i}=\sum_{j=1}^{10}\int_{-10}^{10}|f_{j}(t)-{\bar{f}}_{j}^{i}(t)|dt$ and calculate ${\mathrm{SUP}}_{f}^{i}=\max_{j\in \{1,\ldots ,10\},t\in \tilde{t}}|f_{j}(t)-{\bar{f}}_{j}^{i}(t)|$. We calculate means and 90% quantile‐based intervals for the ISEs, IAEs, and sup‐norm distances across the 1000 simulated data sets.

## 4 Coverage of Credible Intervals

We assess the coverage of the 0.95 credible intervals on simulated data. Table 1 shows the coverage at censored points (which attains 0.93 on large data sets) and at non‐censored points (which attains 0.94 on large data sets). The details of the simulation setup are as follows. We draw 1000 underlying GPs with mean 0 and an exponentiated quadratic covariance function with magnitude 1 and length scale 1. From each of these, we draw a data set by adding $N(0,0.2^{2})$ noise to an equidistant grid of $N_{\text{obs}}$ observations on the interval [−10, 10]. For each data set, we censor observations above the 0.75 quantile so that each data set has a censoring percentage of 25%. We fit the model to each data set and obtain 0.95 credible intervals for each point. The coverage is now computed as the proportion of credible intervals containing the true function value at that time point. It is computed separately for censored points and non‐censored points to show that close‐to‐nominal coverage is obtained at both.

**TABLE 1:** Coverage of 0.95 credible intervals at non‐censored and censored points.

	Coverage	
No. of observations ($N_{\text{obs}}=J_{o}+J_{c}$)	Non‐censored points	Censored points
50	0.92	0.92
100	0.94	0.93
200	0.94	0.93
300	0.94	0.93

## 5 Application: HIV‐1 RNA Viral Load

We now apply our method for value‐censored multi‐level Gaussian process regression to the A5055 data set introduced in Acosta et al. [19]. This data set has previously been analyzed in, for example, Mattos et al. [10], Lachos et al. [11], Wang et al. [20], and Lin and Wang [21]. The data analyzed consists of a total of 365 observations on 44 patients infected with HIV‐1. For each patient, plasma HIV‐1 RNA viral load was measured in copies/mL from blood samples. The blood samples were taken at an irregular number of days, varying from study day 0 to day 205. The median number of observations per subject was nine, the minimum was three, and the maximum was eleven. Thirty‐five (80%) of the subjects had eight or more observations during the study period. Due to a lower detection limit in the measurement process, values below 50 copies/mL were left‐censored. In total, 110 (30%) of the observations were censored with a median number of censorings per subject of two, minimum equal to zero, and maximum equal to eight. 32 (73%) of the subjects had at least one censored observation during their follow‐up time.

**FIGURE 3:**
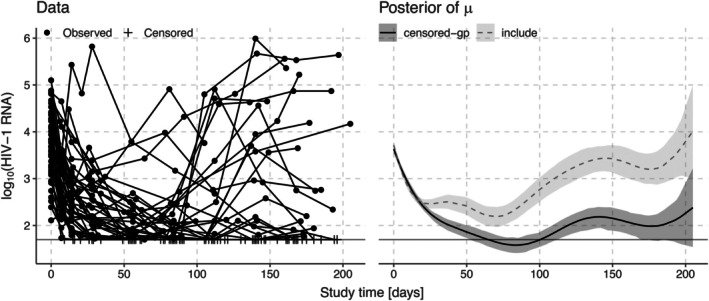
HIV‐1 RNA data set and posterior means and 90% credible intervals for the mean μ when using the method proposed in this paper that correctly accounts for censoring (censored‐GP) or when naively including the censoring values as observations (include). The horizontal line shows the censoring threshold.

**FIGURE 4:**
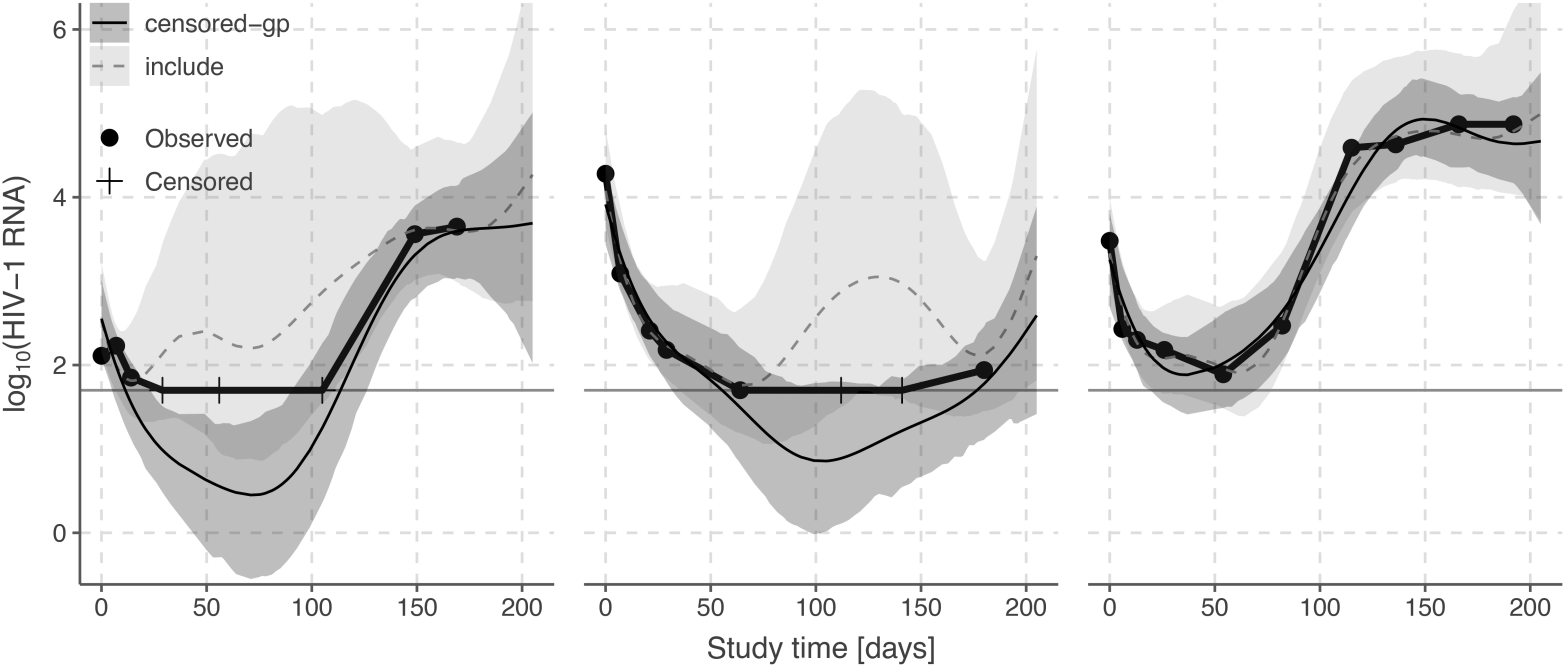
HIV‐1 RNA data (points connected by thick line) for subjects 1 (left panel), 9 (middle panel) and 41 (right panel) and corresponding posterior means and 90% intervals for the latent function $f_{i}$ when using the method proposed in this paper that correctly accounts for censoring (censored‐GP) or when naively including the censoring values as observations (include). The horizontal line shows the censoring threshold.

Figure 3 shows the A5055 data on the $\log_{10}$ scale. The observed data points are marked with circles, and the censored observations are marked with plus signs. The horizontal line shows the lower limit of detection at $\log_{10}50$. We compare posterior means and 90% credible intervals for μ from the include method, which includes all censored observations as if they were regular observed data points, to the posterior distribution from our method censored‐GP, which correctly accounts for censoring. Figure 4 likewise shows posterior means and 90% credible intervals for three of the subject specific curves $f_{1}$, $f_{9}$, and $f_{41}$, again comparing our censored‐GP method to the include method. Naively including the censoring values as observations leads to a large bias in the posterior distributions of μ and f.

## 6 Discussion

In this paper, we have given a procedure for Gaussian process regression in the setting where some of the observations are value‐censored from above, below, or known to be within an interval in the form of an exact analytic expression of the likelihood of the observed data, and a way to directly simulate from the conditional posterior of the latent function. This was done both for the classic single‐curve Gaussian process regression setting and a multi‐level setting with multiple curves around a common mean curve. Simulation studies showed large improvement over naively including or excluding the censored observations and large runtime improvements.

While our expressions have exact and analytic forms, they require numerical evaluations of the multivariate normal CDF and the multivariate truncated normal distribution. Fast numeric algorithms exist [22, 23] when the dimension is below 1000, but future work is required to investigate how these numerical algorithms scale with the proportion of censored observations.

While our proposed method can, in principle, be used for full Bayesian inference using Hamiltonian Monte Carlo, it requires an implementation of the multivariate normal CDF, which is not currently built into the probabilistic programming language Stan. Future work could consider how to do a suitable approximation in Stan of the multivariate normal CDF and its gradient using, for example, importance sampling as in Christoffersen et al. [24]. Additional future work would be to compare the performance of empirical Bayes and full Bayes GP regression for different choices of kernels and priors.

Our simulation studies and examples show the behavior of the posterior mean. Future work might consider the behavior of other summary choices, such as the conditional maximum a posteriori estimate of the latent function f, which maximizes the conditional posterior in Equation (3). This will be possible under the empirical Bayes approach introduced in this paper thanks to the closed‐form expression for the conditional posterior in Equation (3), but it will most likely be difficult to find the maximum a posteriori estimate under the full Bayes approach since a closed‐form expression for the posterior $p(f(\tilde{t})|Y^{o}=y^{o},Y^{c}>y^{c})$ might be hard to find.

## Conflicts of Interest

The authors declare no conflicts of interest.

## Supporting information




**Data S1.** Supporting Information.

## Data Availability

The data that support the findings of this study are openly available in censored‐GP at https://github.com/adamgorm/censored‐gp.
