# Supplementary material for: Gaussian Process Regression for Value‐Censored Functional and Longitudinal Data
Source: Stat Med. 2025 Sep 23;44(20-22):e70277. doi: 10.1002/sim.70277 (PMC12455908; doi:10.1002/sim.70277)
Supplement: Supplementary file 1 — Data S1. Supporting Information. [file SIM-44-0-s001.pdf]

Supplementary material for *Gaussian process regression for value-censored functional and longitudinal data*

Adam Gorm Hoffmann*[1], Claus Thorn Ekstrøm[1], Benjamin Zeymer Christoffersen[2,3], and Andreas Kryger Jensen[1]

[1]Section of Biostatistics, Department of Public Health, University of Copenhagen, Denmark

[2]Division of Robotics, Perception and Learning, KTH Royal Institute of Technology, Stockholm, Sweden

[3]Department of Medical Epidemiology and Biostatistics, Karolinska Institutet, Solna, Sweden

Note: We refer to propositions and equations from the paper.

Proof of Proposition 1

The model implies

$$\begin{pmatrix} \boldsymbol{Y}^o \\ \boldsymbol{Y}^c \end{pmatrix} \mid \boldsymbol{\Theta} \sim N\left(\begin{pmatrix} 0_{J_o} \\ 0_{J_c} \end{pmatrix}, \begin{pmatrix} \boldsymbol{K}_{\boldsymbol{\theta}}(\boldsymbol{t}^o, \boldsymbol{t}^o) + \sigma^2 \boldsymbol{I}_{J_o} & \boldsymbol{K}_{\boldsymbol{\theta}}(\boldsymbol{t}^o, \boldsymbol{t}^c) \\ \boldsymbol{K}_{\boldsymbol{\theta}}(\boldsymbol{t}^c, \boldsymbol{t}^o) & \boldsymbol{K}_{\boldsymbol{\theta}}(\boldsymbol{t}^c, \boldsymbol{t}^c) + \sigma^2 \boldsymbol{I}_{J_c} \end{pmatrix} \right)$$

and thus we get by the rules of conditioning in the multivariate normal distribution that

$$\boldsymbol{Y}^c \mid \boldsymbol{Y}^o = \boldsymbol{y}^o, \boldsymbol{\Theta} \sim N(\boldsymbol{\xi}_{c|o}, \boldsymbol{\Sigma}_{c|o})$$

*Corresponding author. adam.hoffmann@sund.ku.dk, Section of Biostatistics, Department of Public Health, University of Copenhagen. Øster Farimagsgade 5. DK-1353 Copenhagen. Denmark.

with

$$\boldsymbol{\xi}_{c|o} = \boldsymbol{K}_{\boldsymbol{\theta}}(\boldsymbol{t}^c, \boldsymbol{t}^o)[\boldsymbol{K}_{\boldsymbol{\theta}}(\boldsymbol{t}^o, \boldsymbol{t}^o) + \sigma^2 \boldsymbol{I}_{J_o}]^{-1} \boldsymbol{y}^o,$$
$$\boldsymbol{\Sigma}_{c|o} = \boldsymbol{K}_{\boldsymbol{\theta}}(\boldsymbol{t}^c, \boldsymbol{t}^c) + \sigma^2 \boldsymbol{I}_{J_c} - \boldsymbol{K}_{\boldsymbol{\theta}}(\boldsymbol{t}^c, \boldsymbol{t}^o)[\boldsymbol{K}_{\boldsymbol{\theta}}(\boldsymbol{t}^o, \boldsymbol{t}^o) + \sigma^2 \boldsymbol{I}_{J_o}]^{-1} \boldsymbol{K}_{\boldsymbol{\theta}}(\boldsymbol{t}^o, \boldsymbol{t}^c).$$

Writing $\boldsymbol{\Sigma}_o := \boldsymbol{K}_{\boldsymbol{\theta}}(\boldsymbol{t}^o, \boldsymbol{t}^o) + \sigma^2 \boldsymbol{I}_{J_o}$, we thus get the likelihood

$$\begin{aligned} p(\boldsymbol{Y}^o = \boldsymbol{y}^o, \boldsymbol{Y}^c > \boldsymbol{y}^c \mid \boldsymbol{\Theta}) &= p(\boldsymbol{Y}^o = \boldsymbol{y}^o \mid \boldsymbol{\Theta}) p(\boldsymbol{Y}^c > \boldsymbol{y}^c \mid \boldsymbol{Y}^o = \boldsymbol{y}^o, \boldsymbol{\Theta}) \\ &= \phi(\boldsymbol{y}^o; \boldsymbol{0}_{J_o}, \boldsymbol{\Sigma}_o) \Phi(-\boldsymbol{y}^c; -\boldsymbol{\xi}_{c|o}, \boldsymbol{\Sigma}_{c|o}), \end{aligned}$$

as desired.

Proof of Equation (3)

A direct calculation gives that

$$\begin{aligned} &p(\boldsymbol{f}(\tilde{\boldsymbol{t}}) \mid \boldsymbol{Y}^o = \boldsymbol{y}^o, \boldsymbol{Y}^c > \boldsymbol{y}^c, \boldsymbol{\Theta}) \\ &\quad = \frac{p(\boldsymbol{f}(\tilde{\boldsymbol{t}}), \boldsymbol{Y}^o = \boldsymbol{y}^o, \boldsymbol{Y}^c > \boldsymbol{y}^c \mid \boldsymbol{\Theta})}{p(\boldsymbol{Y}^o = \boldsymbol{y}^o, \boldsymbol{Y}^c > \boldsymbol{y}^c \mid \boldsymbol{\Theta})} \\ &\quad = \frac{p(\boldsymbol{f}(\tilde{\boldsymbol{t}}), \boldsymbol{Y}^o = \boldsymbol{y}^o \mid \boldsymbol{\Theta}) p(\boldsymbol{Y}^c > \boldsymbol{y}^c \mid \boldsymbol{Y}^o = \boldsymbol{y}^o, \boldsymbol{f}(\tilde{\boldsymbol{t}}), \boldsymbol{\Theta})}{p(\boldsymbol{Y}^o = \boldsymbol{y}^o \mid \boldsymbol{\Theta}) p(\boldsymbol{Y}^c > \boldsymbol{y}^c \mid \boldsymbol{Y}^o = \boldsymbol{y}^o, \boldsymbol{\Theta})} \\ &\quad = p(\boldsymbol{f}(\tilde{\boldsymbol{t}}) \mid \boldsymbol{Y}^o = \boldsymbol{y}^o, \boldsymbol{\Theta}) \frac{p(\boldsymbol{Y}^c > \boldsymbol{y}^c \mid \boldsymbol{Y}^o = \boldsymbol{y}^o, \boldsymbol{f}(\tilde{\boldsymbol{t}}), \boldsymbol{\Theta})}{p(\boldsymbol{Y}^c > \boldsymbol{y}^c \mid \boldsymbol{Y}^o = \boldsymbol{y}^o, \boldsymbol{\Theta})} \\ &\quad = \phi(\boldsymbol{f}(\tilde{\boldsymbol{t}}) \mid \boldsymbol{\xi}_{f|o}, \boldsymbol{\Sigma}_{f|o}) \frac{\Phi(-\boldsymbol{y}^c; -\boldsymbol{\xi}_{c|o,f}, \boldsymbol{\Sigma}_{c|o,f})}{\Phi(-\boldsymbol{y}^c; -\boldsymbol{\xi}_{c|o}, \boldsymbol{\Sigma}_{c|o})}. \end{aligned}$$

The expression for the means and covariances are given in Equation (4) as a direct application of conditioning in the multivariate normal distribution.

Proof of Proposition 2

The model gives that

$$\begin{pmatrix} \boldsymbol{f}(\tilde{\boldsymbol{t}}) \\ \boldsymbol{Y}^c \\ \boldsymbol{Y}^o \end{pmatrix} \mid \boldsymbol{\Theta} \sim N\left(\begin{pmatrix} \mathbf{0}_{J_p} \\ \mathbf{0}_{J_c} \\ \mathbf{0}_{J_o} \end{pmatrix}, \begin{pmatrix} \boldsymbol{\Sigma}_f & \boldsymbol{\Sigma}_{fc} & \boldsymbol{\Sigma}_{fo} \\ \boldsymbol{\Sigma}_{cf} & \boldsymbol{\Sigma}_c & \boldsymbol{\Sigma}_{co} \\ \boldsymbol{\Sigma}_{of} & \boldsymbol{\Sigma}_{oc} & \boldsymbol{\Sigma}_o \end{pmatrix}\right)$$

where

$$\begin{aligned} \boldsymbol{\Sigma}_f &= \boldsymbol{K}_{\boldsymbol{\theta}}(\tilde{\boldsymbol{t}}, \tilde{\boldsymbol{t}}) \\ \boldsymbol{\Sigma}_c &= \boldsymbol{K}_{\boldsymbol{\theta}}(\boldsymbol{t}^c, \boldsymbol{t}^c) + \sigma^2 \boldsymbol{I}_{J_c} \\ \boldsymbol{\Sigma}_o &= \boldsymbol{K}_{\boldsymbol{\theta}}(\boldsymbol{t}^o, \boldsymbol{t}^o) + \sigma^2 \boldsymbol{I}_{J_o} \\ \boldsymbol{\Sigma}_{fc} &= \boldsymbol{K}_{\boldsymbol{\theta}}(\tilde{\boldsymbol{t}}, \boldsymbol{t}^c) \\ \boldsymbol{\Sigma}_{fo} &= \boldsymbol{K}_{\boldsymbol{\theta}}(\tilde{\boldsymbol{t}}, \boldsymbol{t}^o) \\ \boldsymbol{\Sigma}_{co} &= \boldsymbol{K}_{\boldsymbol{\theta}}(\boldsymbol{t}^c, \boldsymbol{t}^o). \end{aligned}$$

Conditioning in the multivariate normal distribution then gives that

$$\begin{pmatrix} \boldsymbol{f}(\tilde{\boldsymbol{t}}) \\ \boldsymbol{Y}^c \end{pmatrix} \mid \boldsymbol{Y}^o = \boldsymbol{y}^o, \boldsymbol{\Theta} \sim N\left(\begin{pmatrix} \boldsymbol{\xi}_{f|o} \\ \boldsymbol{\xi}_{c|o} \end{pmatrix}, \begin{pmatrix} \boldsymbol{\Sigma}_{f|o} & \boldsymbol{\Sigma}_{fc|o} \\ \boldsymbol{\Sigma}_{cf|o} & \boldsymbol{\Sigma}_{c|o} \end{pmatrix}\right)$$

where

$$\begin{aligned} \boldsymbol{\xi}_{f|o} &= \boldsymbol{\Sigma}_{fo} \boldsymbol{\Sigma}_o^{-1} \boldsymbol{y}^o \\ \boldsymbol{\Sigma}_{f|o} &= \boldsymbol{\Sigma}_f - \boldsymbol{\Sigma}_{fo} \boldsymbol{\Sigma}_o^{-1} \boldsymbol{\Sigma}_{of} \\ \boldsymbol{\Sigma}_{fc|o} &= \boldsymbol{\Sigma}_{fc} - \boldsymbol{\Sigma}_{fo} \boldsymbol{\Sigma}_o^{-1} \boldsymbol{\Sigma}_{oc}. \end{aligned}$$

We can now use Arellano-Valle et al. [1, Section 2.3] where, always working conditionally on $\boldsymbol{Y}^o = \boldsymbol{y}^o, \boldsymbol{\Theta}$, we let $\boldsymbol{V} = \boldsymbol{f}(\tilde{\boldsymbol{t}})$ and $\boldsymbol{U} = \boldsymbol{Y}^c$ with the selection subset $C = \{\boldsymbol{x} \in \mathbb{R}^{J_c} : \boldsymbol{x} > \boldsymbol{y}^c\}$ and thus get the desired representation of the distribution from Arellano-Valle et al. [1, (15)].

Proof of Proposition 3

It follows from the model specification in Equation (5) that

$$\begin{pmatrix} \boldsymbol{Y}^c \\ \boldsymbol{Y}^o \end{pmatrix} \mid \boldsymbol{\Theta} \sim N\left(\begin{pmatrix} \mathbf{0}_{J_c} \\ \mathbf{0}_{J_o} \end{pmatrix}, \begin{pmatrix} \boldsymbol{\Omega}_c & \boldsymbol{\Omega}_{co} \\ \boldsymbol{\Omega}_{oc} & \boldsymbol{\Omega}_o \end{pmatrix} \right)$$

with the covariance matrices given in Equation (6). Conditioning in the multivariate normal distribution then gives

$$\boldsymbol{Y}^c \mid \boldsymbol{Y}^o = \boldsymbol{y}^o, \boldsymbol{\Theta} \sim N(\boldsymbol{\nu}_{c|o}, \boldsymbol{\Omega}_{c|o})$$

where

$$\begin{aligned} \boldsymbol{\nu}_{c|o} &= \boldsymbol{\Omega}_{co}\boldsymbol{\Omega}_o^{-1}\boldsymbol{y}^o \\ \boldsymbol{\Omega}_{c|o} &= \boldsymbol{\Omega}_c - \boldsymbol{\Omega}_{co}\boldsymbol{\Omega}_o^{-1}(\boldsymbol{\Omega}_{co})^T. \end{aligned}$$

Using this, we can express the likelihood

$$\begin{aligned} &p(\boldsymbol{Y}^o = \boldsymbol{y}^o, \boldsymbol{Y}^c > \boldsymbol{y}^c \mid \boldsymbol{\Theta}) \\ &\qquad = p(\boldsymbol{Y}^o = \boldsymbol{y}^o \mid \boldsymbol{\Theta}) p(\boldsymbol{Y}^c > \boldsymbol{y}^c \mid \boldsymbol{Y}^o = \boldsymbol{y}^o, \boldsymbol{\Theta}) \\ &\qquad = \phi(\boldsymbol{y}^o; \mathbf{0}_{J_o}, \boldsymbol{\Omega}_o)\Phi(-\boldsymbol{y}^c; -\boldsymbol{\nu}_{c|o}, \boldsymbol{\Omega}_{c|o}), \end{aligned}$$

as desired.

Proof of Equation (7)

The same calculation as in the proof of Equation (3), but with $(\boldsymbol{\mu}(\tilde{\boldsymbol{t}}), \boldsymbol{\eta}'(\tilde{\boldsymbol{t}}))$ instead of $\boldsymbol{f}(\tilde{\boldsymbol{t}})$, yields that

$$\begin{aligned} &p(\boldsymbol{\mu}(\tilde{\boldsymbol{t}}), \boldsymbol{\eta}'(\tilde{\boldsymbol{t}}) \mid \boldsymbol{Y}^o = \boldsymbol{y}^o, \boldsymbol{Y}^c > \boldsymbol{y}^c, \boldsymbol{\Theta}) \\ &\qquad = p(\boldsymbol{\mu}(\tilde{\boldsymbol{t}}), \boldsymbol{\eta}'(\tilde{\boldsymbol{t}}) \mid \boldsymbol{Y}^o = \boldsymbol{y}^o, \boldsymbol{\Theta}) \frac{p(\boldsymbol{Y}^c > \boldsymbol{y}^c \mid \boldsymbol{Y}^o = \boldsymbol{y}^o, \boldsymbol{\mu}(\tilde{\boldsymbol{t}}), \boldsymbol{\eta}'(\tilde{\boldsymbol{t}}), \boldsymbol{\Theta})}{p(\boldsymbol{Y}^c > \boldsymbol{y}^c \mid \boldsymbol{Y}^o = \boldsymbol{y}^o, \boldsymbol{\Theta})} \\ &\qquad = \phi(\boldsymbol{\mu}(\tilde{\boldsymbol{t}}), \boldsymbol{\eta}'(\tilde{\boldsymbol{t}}) \mid \boldsymbol{\nu}_{\mu,\eta'|o}, \boldsymbol{\Omega}_{\mu,\eta'|o}) \frac{\Phi(-\boldsymbol{y}^c; -\boldsymbol{\nu}_{c|\mu,\eta',o}, \boldsymbol{\Omega}_{c|\mu,\eta',o})}{\Phi(-\boldsymbol{y}^c; -\boldsymbol{\nu}_{c|o}, \boldsymbol{\Omega}_{c|o})}. \end{aligned}$$

The model specification in Equation (5) implies that

$$\begin{pmatrix} \boldsymbol{Y}^c \\ \boldsymbol{\mu}(\tilde{\boldsymbol{t}}) \\ \boldsymbol{\eta}'(\tilde{\boldsymbol{t}}) \\ \boldsymbol{Y}^o \end{pmatrix} \mid \boldsymbol{\Theta} \sim N \left(\begin{pmatrix} \mathbf{0}_{J_c} \\ \mathbf{0}_{J_p} \\ \mathbf{0}_{(n-1)J_p} \\ \mathbf{0}_{J_o} \end{pmatrix}, \begin{pmatrix} \boldsymbol{\Omega}_c & \boldsymbol{\Omega}_{c\mu} & \boldsymbol{\Omega}_{c\eta'} & \boldsymbol{\Omega}_{co} \\ \boldsymbol{\Omega}_{\mu c} & \boldsymbol{\Omega}_{\mu} & \mathbf{0}_{J_p,(n-1)J_p} & \boldsymbol{\Omega}_{\mu o} \\ \boldsymbol{\Omega}_{\eta' c} & \mathbf{0}_{(n-1)J_p,J_p} & \boldsymbol{\Omega}_{\eta'} & \boldsymbol{\Omega}_{\eta' o} \\ \boldsymbol{\Omega}_{oc} & \boldsymbol{\Omega}_{o\mu} & \boldsymbol{\Omega}_{o\eta'} & \boldsymbol{\Omega}_{o} \end{pmatrix} \right).$$

The expressions in Equation (8) follow by conditioning in the multivariate normal distribution to get $\boldsymbol{\mu}(\tilde{\boldsymbol{t}}), \boldsymbol{\eta}'(\tilde{\boldsymbol{t}}), \boldsymbol{Y}^c \mid \boldsymbol{Y}^o, \boldsymbol{\Theta}$ and $\boldsymbol{Y}^c \mid \boldsymbol{\mu}(\tilde{\boldsymbol{t}}), \boldsymbol{\eta}'(\tilde{\boldsymbol{t}}), \boldsymbol{Y}^o, \boldsymbol{\Theta}$.

Proof of Proposition 4

By conditioning on $\boldsymbol{Y}^o = \boldsymbol{y}^o$ in the joint distribution at the bottom of the proof of Equation (7), we get that

$$\begin{pmatrix} \boldsymbol{Y}^c \\ \boldsymbol{\mu}(\tilde{\boldsymbol{t}}) \\ \boldsymbol{\eta}'(\tilde{\boldsymbol{t}}) \end{pmatrix}$$

is normally distributed with covariance matrix

$$\begin{pmatrix} \boldsymbol{\Omega}_c & \boldsymbol{\Omega}_{c\mu} & \boldsymbol{\Omega}_{c\eta'} \\ \boldsymbol{\Omega}_{\mu c} & \boldsymbol{\Omega}_{\mu} & \mathbf{0} \\ \boldsymbol{\Omega}_{\eta' c} & \mathbf{0} & \boldsymbol{\Omega}_{\eta'} \end{pmatrix} - \begin{pmatrix} \boldsymbol{\Omega}_{co} \\ \boldsymbol{\Omega}_{\mu o} \\ \boldsymbol{\Omega}_{\eta' o} \end{pmatrix} \boldsymbol{\Omega}_o^{-1} \begin{pmatrix} \boldsymbol{\Omega}_{oc} & \boldsymbol{\Omega}_{o\mu} & \boldsymbol{\Omega}_{o\eta'} \end{pmatrix}.$$

In particular, this gives that $\mathrm{Cov}((\boldsymbol{\mu}(\tilde{\boldsymbol{t}})^T, \boldsymbol{\eta}'(\tilde{\boldsymbol{t}})^T)^T, \boldsymbol{Y}^c \mid \boldsymbol{Y}^o = \boldsymbol{y}^o, \boldsymbol{\Theta}) = \boldsymbol{\Omega}_{(\mu,\eta')c|o}$ as expressed in Equation (9). The rest of the covariances in Equation (9) follow directly from the model specification in Equation (5).

As earlier, we can now use Arellano-Valle et al. [1, Section 2.3] where, working conditionally on $\boldsymbol{Y}^o = \boldsymbol{y}^o, \boldsymbol{\Theta}$, we let $\boldsymbol{V} = (\boldsymbol{\mu}(\tilde{\boldsymbol{t}})^T, \boldsymbol{\eta}'(\tilde{\boldsymbol{t}})^T)^T$ and $\boldsymbol{U} = \boldsymbol{Y}^c$ with the selection subset $C = \{\boldsymbol{x} \in \mathbb{R}^{J_c} : \boldsymbol{x} > \boldsymbol{y}^c\}$ and thus get the desired representation of the distribution from Arellano-Valle et al. [1, (15)].

Additional simulation results

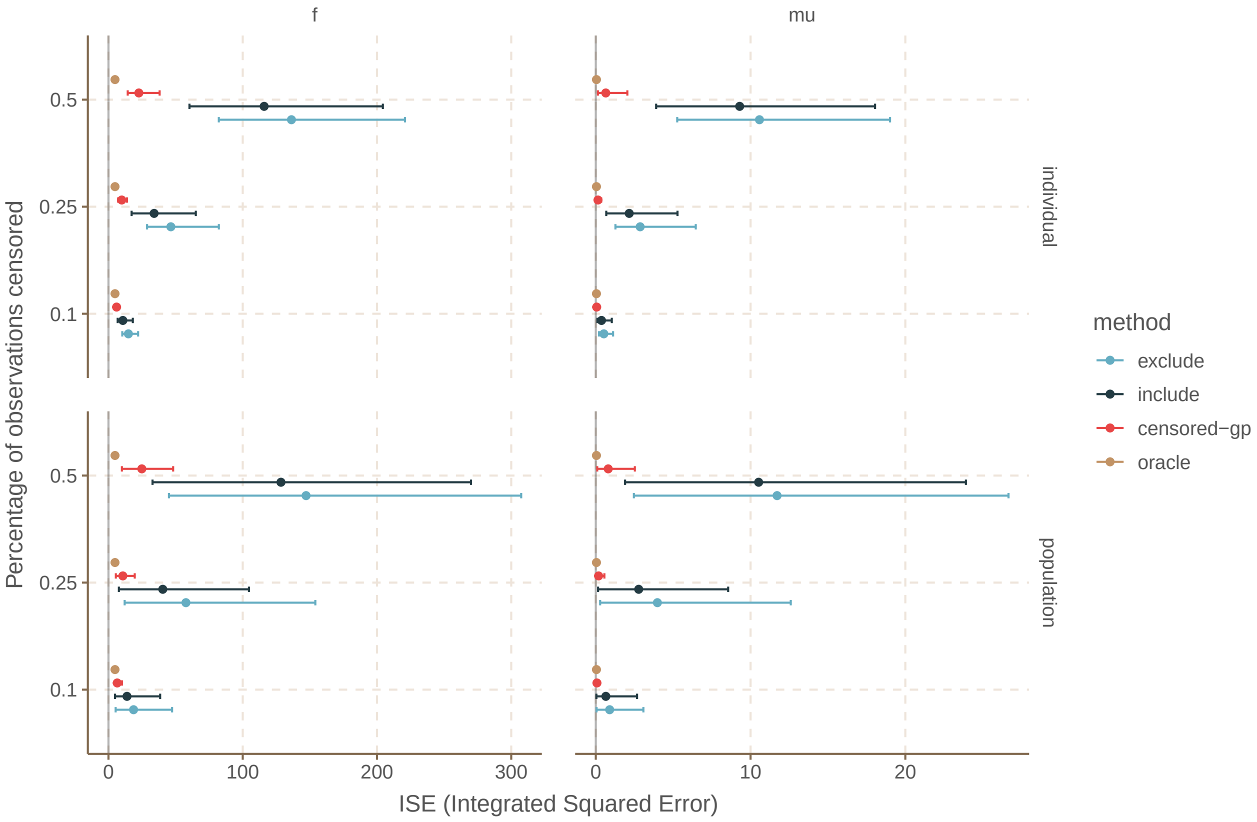

Figure 1: Similar to Figure 2. ISE based on simulation observed at $n_{obs} = 51$ equidistant time points.

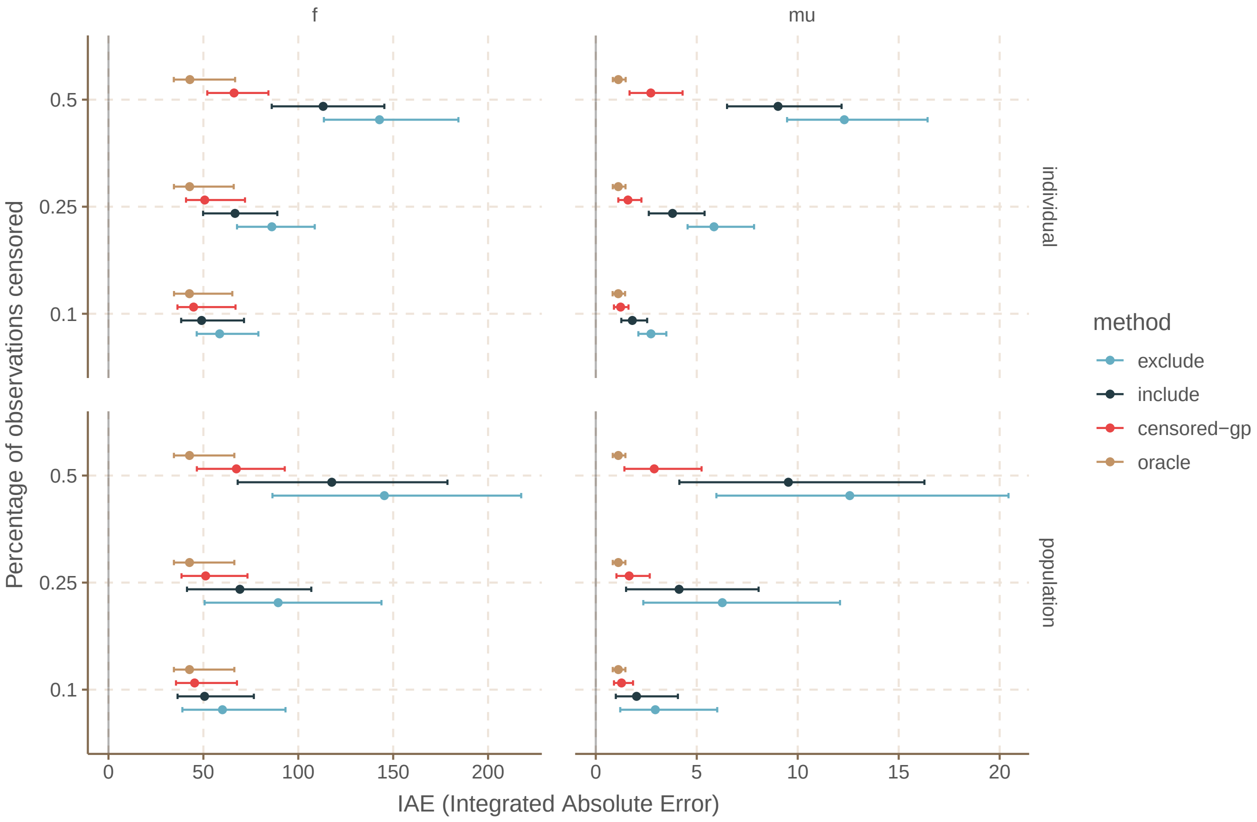

Figure 2: Similar to Figure 2. IAE based on simulation observed at $n_{obs} = 26$ equidistant time points.

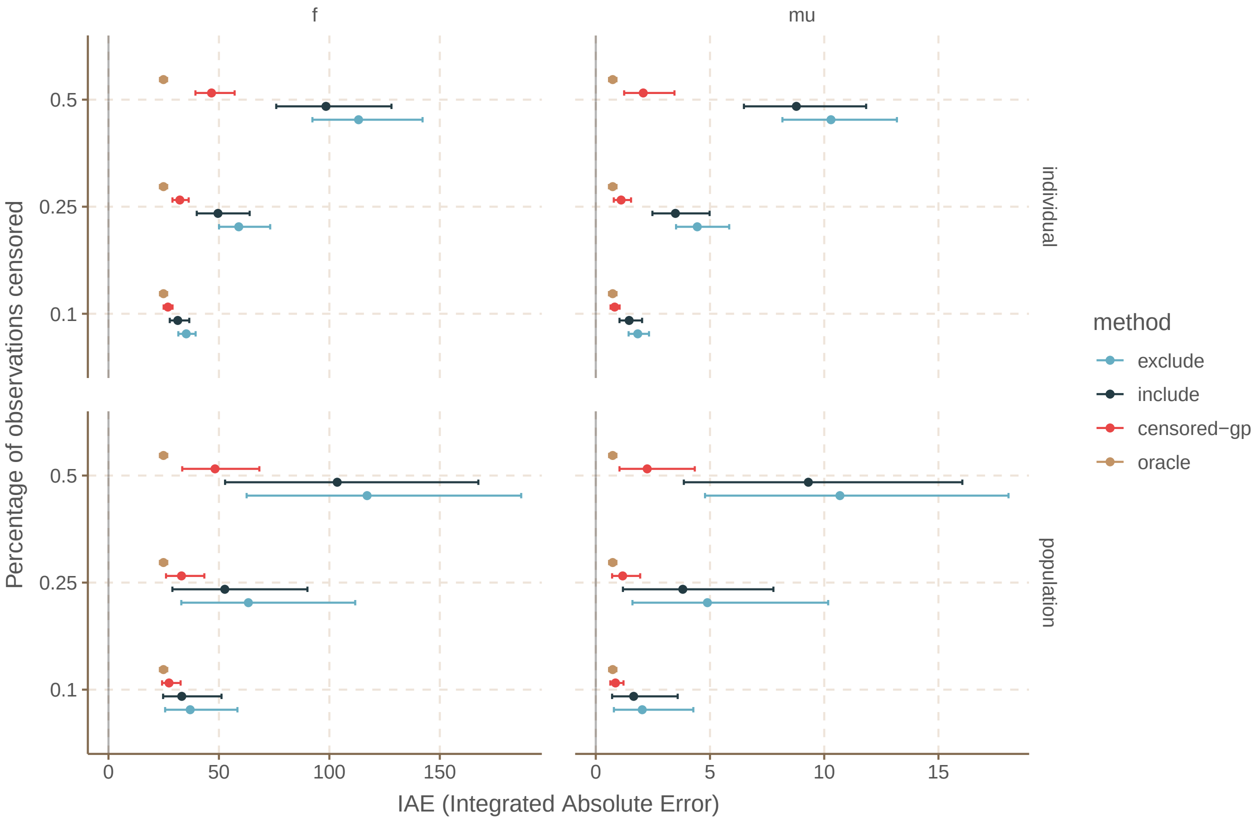

Figure 3: Similar to Figure 2. IAE based on simulation observed at $n_{obs} = 51$ equidistant time points.

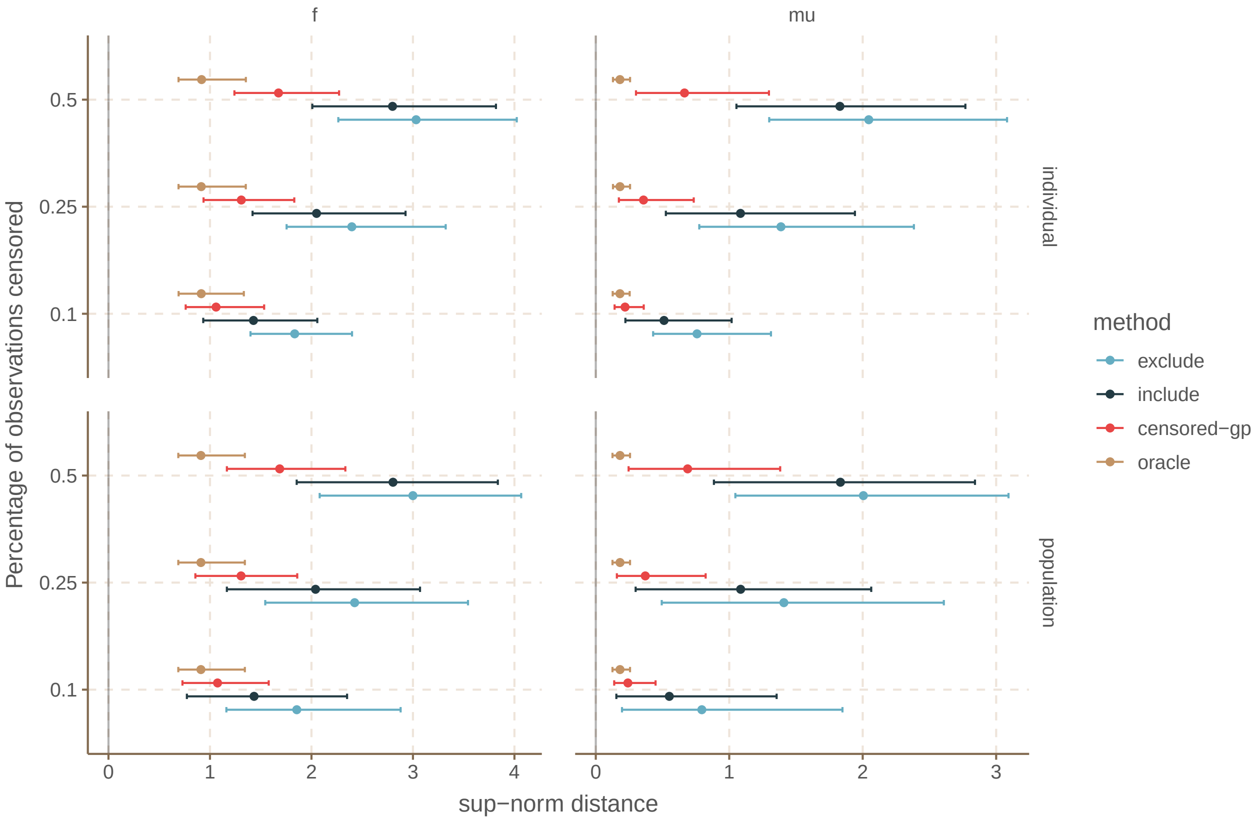

Figure 4: Similar to Figure 2. sup-norm distance based on simulation observed at $n_{obs} = 26$ equidistant time points.

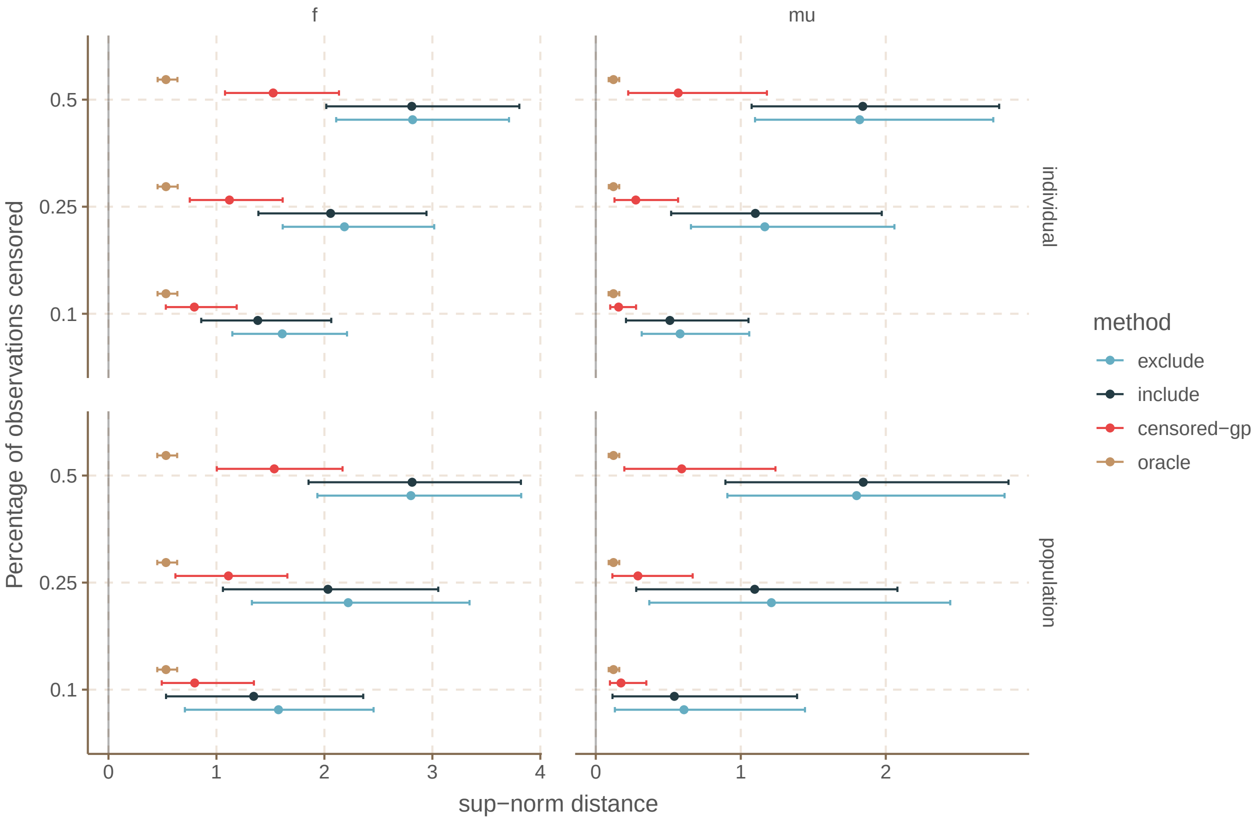

Figure 5: Similar to Figure 2. sup-norm distance based on simulation observed at $n_{obs} = 51$ equidistant time points.

Comparing runtime to naive full Bayes implementation

As mentioned in the paper, it is possible to do full Bayesian inference by implementing the posterior density $p(\boldsymbol{f}(\boldsymbol{t}), \boldsymbol{\Theta} \mid \boldsymbol{Y}^o, \boldsymbol{Y}^c > \boldsymbol{y}^c) \propto p(\boldsymbol{Y}^o, \boldsymbol{Y}^c > \boldsymbol{y}^c \mid \boldsymbol{f}(\boldsymbol{t}), \boldsymbol{\Theta}) p(\boldsymbol{f}(\boldsymbol{t}) \mid \boldsymbol{\Theta}) p(\boldsymbol{\Theta})$ in a probabilistic programming language such as Stan. Since this requires including the $J_o + J_c + J_p$ function values $\boldsymbol{f}(\boldsymbol{t}) = \{\boldsymbol{f}(\boldsymbol{t}^o), \boldsymbol{f}(\boldsymbol{t}^c), \boldsymbol{f}(\tilde{\boldsymbol{t}})\}$ as parameters and doing full MCMC with no conjugacy, it leads to much larger runtimes for large data sets as seen in Figure 6.

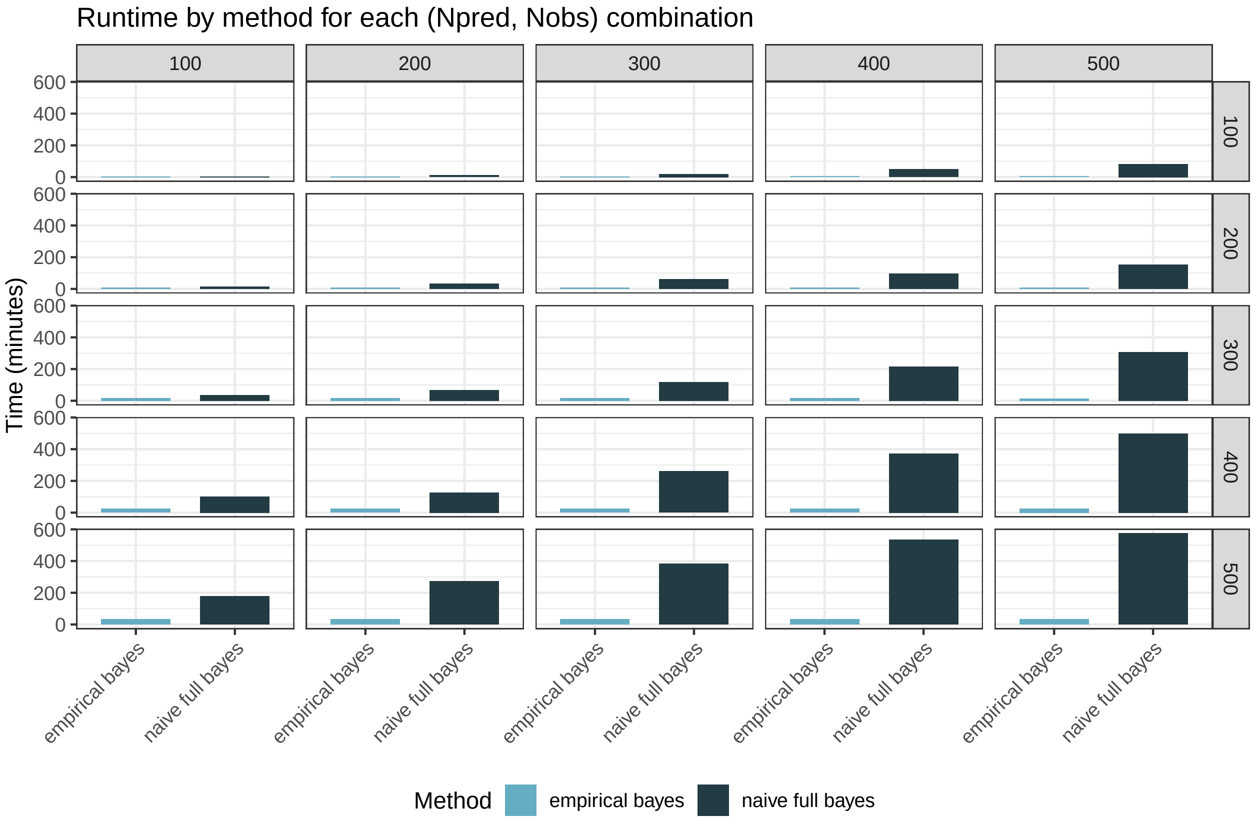

Figure 6: Runtime when comparing an empirical Bayes implementation using Proposition 2 to a naive full Bayes implementation on simulated data when fit to observations at Nobs ($J_o + J_c$) equidistant time points (panels varying horizontally) and predicting at Npred (J_p) equidistant time points (panels varying vertically). The empirical Bayes implementation is 1 to 19 times faster than the naive full Bayes implementation, being much faster when Nobs or Npred are large and not much faster when they are small.

References

[1] Arellano-Valle, R. B., M. D. Branco, and M. G. Genton (2006). A unified view on skewed distributions arising from selections. *Canadian Journal of Statistics 34*(4), 581–601.
